# PotatoGuardNet: a refined deep learning framework for potato leaf disease detection

**DOI:** 10.3389/fpls.2026.1720276

**Published:** 2026-01-30

**Authors:** Marriam Nawaz, Ali Javed, Abdul Khader Jilani Saudagar

**Affiliations:** 1 Department of Software Engineering, University of Engineering and Technology-Taxila, Taxila, Pakistan; 2 Information Systems Department, College of Computer and Information Sciences, Imam Mohammad Ibn Saud Islamic University (IMSIU), Riyadh, Saudi Arabia

**Keywords:** classification, computer vision, deep learning, Faster-RCNN, InceptionResNet-V2, potato diseases

## Abstract

**Introduction:**

The potato is one of the most consumed vegetable crops worldwide. However, the environmental changes and various crop diseases have a significant impact on potato production, indicating severe damage to yield quality and quantity. Farmers mostly employ manual disease classification methods in agriculture, which have limitations in detecting subtle disease symptoms, are time-intensive, and often necessitate specialized expertise, which may not be accessible in all farming communities. Therefore, automated systems are designed for accurate and rapid disease classification, mitigating the risks of misdiagnoses and delayed treatments. However, differences in the size, mass, and structure of the diseased areas of potato leaf diseases, combined with complex environmental conditions, complicate the effective identification of these diseases.

**Methods:**

Therefore, to address the existing issues, we propose an improved deep learning approach, namely the PotatoGuardNet, which is an Inception-ResNet-V2-based Faster-RCNN model, for locating and classifying various potato leaf diseases. Precisely, the InceptionResNet-V2 approach is employed as the base network to capture the visual attributes of the samples, which are later recognized and classified by the 2-stage detector of the Faster-RCNN model.

**Results:**

The model is tested on huge and complex data samples of potato plants from the PlantVillage dataset and reported a classification accuracy of 99.41%, along with an mAP of 0.9556. Further, the core working of the proposed method is evaluated by generating the heatmaps to show its explanatory power.

**Discussion:**

Extensive experiments and comparative analyses against several recent state-of-the-art approaches confirm the effectiveness and reliability of PotatoGuardNet for potato leaf disease detection. The results demonstrate that the proposed framework successfully captures disease-specific visual patterns and provides accurate localization and classification, indicating its potential for practical deployment in automated agricultural disease monitoring systems.

## 1 Introduction

In compliance with a recent analysis conducted by the Food and Agriculture Organization (FAO) of the US, it is predicted that the human population will rise exponentially worldwide by 2050, leading to a total of 9.1 billion (Bruinsma, 2009). While this is happening, the expansion of nutrient levels is hampered by the shrinking amount of fields and a shortage of clean water. There is a critical requirement to increase the sustainability of agriculture while utilizing the smallest amount of land in developing regions to satisfy the needs of people. Conversely, the yield and nutritional value of food pointedly decay as a result of various agricultural anomalies. Meanwhile, these diseases tend to lower revenue from agriculture and increase costs of living, so rapid identification of these plant infections is decisive. Such results could lead to economic uncertainty in the whole marketplace. Furthermore, major food crop irregularities can collapse production and result in malnourishment in a nation, especially among emerging economies with limited resources. Planting studies are most likely passed out beside the support of subject matter experts, but this is a challenging and exhausting operation. Furthermore, these techniques for plant inspection have not proven to be highly reliable, and assessing each plant individually is quite challenging (Pantazi et al., 2019). Consequently, it is decisive to diagnose various plant diseases accurately and quickly to prevent producers from resorting to expensive treatment methods and to expedite food production (Zhu et al., 2025). To address the aforementioned issues with traditional processes, the scientific community is directing its efforts toward the development of computerized methods for diagnosing and identifying plant ailments (Wolfenson, 2013).

While there are numerous crops, including tomatoes, onions, and cucumbers, amongst others, the potato stands out as a widely used vegetable worldwide. Potato is ranked third in terms of worldwide agricultural production after grains like wheat and rice. Over a billion individuals around the world consider potatoes a staple in their daily meals. Each year, over 300,000 metric tons are grown globally, providing critical nutrition and calorie resources to people (Chen et al., 2022; Sangar and Rajasekar, 2025). In addition to making a substantial contribution to global food intake, potatoes are also a common source of raw materials for the food processing sector. Every year, potatoes are cultivated worldwide, with China, India, and Russia being among the top three producers (Elnaggar et al., 2018). Potatoes hold immense significance globally, serving as a cornerstone of nutrition and food security for over a billion people. As the third most widely produced crop worldwide, after wheat and rice, they provide a cost-effective source of essential nutrients and calories, playing a pivotal role in combating hunger and malnutrition. Potatoes are not only a dietary staple but also a driver of economic growth, supporting livelihoods in regions where they are cultivated. Their adaptability to diverse climates and resilience in challenging agricultural environments make them a dependable resource for food production. Furthermore, the versatility of potatoes in culinary applications contributes to their cultural and gastronomic importance, enriching diets across the globe.

Various diseases can significantly harm potato yields, posing a substantial impact on global agriculture and food security. Diseases like late blight and early blight are major threats that can lead to crop losses, reduce quality, and increase production costs (Reis and Turk, 2024). Early blight is caused by the fungal pathogen *Alternaria solani* and is characterized by small, dark brown lesions with concentric rings that typically appear on older leaves. These lesions can expand rapidly, leading to premature leaf senescence and reduced photosynthetic capacity. Late blight, caused by the oomycete *Phytophthora infestans*, is a highly destructive disease that produces water-soaked lesions which quickly turn brown or black, often accompanied by white fungal growth under humid conditions. Late blight can spread rapidly under favorable weather conditions and is capable of destroying entire potato fields within a short period. Both diseases pose a significant economic threat to potato production worldwide, resulting in substantial yield losses, increased management costs, and heavy reliance on fungicides. These diseases not only affect the quantity of potatoes harvested but also threaten their nutritional value and suitability for consumption. Such yield reductions have far-reaching consequences, especially in regions where potatoes are an alimentary staple. The economic impact is substantial, as decreased yields can lead to higher potato prices and affect the livelihoods of farmers and communities heavily dependent on potato cultivation. Addressing and managing these diseases through research, sustainable farming practices, and disease-resistant potato varieties is critical for ensuring a stable and nutritious food supply for millions of people worldwide (Singh et al., 2024). According to a report, the biggest barrier to the rate of potato production is the widespread incidence of numerous diseases, most of which arise from the leaf surfaces of the potato plants and induce a drop in productivity ranging from 9% to 11% yearly (Sardogan et al., 2018). The academic community first applied techniques from the domains of the natural sciences and cellular biology to investigate potato plant leaf problems (Sankaran et al., 2010; Dinh et al., 2020). These techniques, however, have a considerable amount of computational burden and necessitate a high level of expertise (Ferentinos, 2018). Since individuals with limited resources produce most of the agriculture, such expensive technologies are not appropriate for them (Patil and Chandavale, 2015). Machine learning (ML) techniques, including Support Vector Machine (SVM), K-Nearest Neighbors (KNN), and others, have immense significance in agricultural image analysis. They enable the automation and precision of various critical tasks, such as crop disease detection, yield prediction, and soil quality assessment. These algorithms empower farmers with data-driven insights, optimizing resource allocation and promoting sustainable practices, thus enhancing food security and environmental conservation. However, it’s crucial to acknowledge their limitations, including the need for substantial labeled datasets, potential bias in training data, and computational requirements, which can be a barrier to adoption for small-scale farmers with limited resources (Ngugi et al., 2024). Additionally, ML models may not always generalize well across diverse agricultural conditions and may require continual adaptation and monitoring to maintain their effectiveness.

DL has addressed several challenges faced by traditional ML methods in image analysis and various domains. DL frameworks are making significant developments in agriculture as well by leveraging their image analysis capabilities. Through Convolutional Neural Networks (CNNs) (Roska and Chua, 1993) and Recurrent Neural Networks (RNNs) (Zaremba et al., 2014), DL models can process vast amounts of agricultural data, such as satellite imagery, drone-captured photos, and sensor data (Salakhutdinov and Hinton, 2009). These models excel in tasks like crop monitoring, disease detection, yield prediction, and soil analysis, enabling farmers to make data-driven decisions for optimized resource allocation and sustainable practices (Yuan and Zhang, 2016). Additionally, DL models can handle vast amounts of unstructured data efficiently, making them well-suited for big data applications (Szegedy et al., 2015). However, DL models typically require large datasets and substantial computational resources for training, which can be limiting factors in some scenarios (Vedaldi and Zisserman, 2016). Nevertheless, their capacity to automatically extract complex features and generalize well to diverse datasets has revolutionized fields such as natural language understanding and computer vision (Szegedy et al., 2015).

Extensive work has been proposed by scientists for the prompt and effective identification of diseases related to the potato plant via using the conventional ML and DL approaches; however, these techniques are facing several challenges, like suffering from model overfitting, especially when dealing with small or imbalanced datasets, which leads to poor generalization to new or unseen disease instances. Interpretability and explainability of existing models are also problematic, making it difficult for farmers and experts to comprehend how the model makes decisions. Furthermore, the computational demands of training and deploying deep networks are prohibitive for small-scale farmers or regions with limited access to high-performance computing resources. Lastly, these models struggle with environmental variability and do not adapt well to changing weather conditions or diverse field conditions, which can affect their robustness and reliability in practical agricultural applications. Another challenge is the correct recognition of potato leaf diseases at the starting phases, as there exists an extensive structural similarity in the healthy and unhealthy regions of leaves (Paul et al., 2020). Analysis shows that very little work has been proposed by employing object detection approaches for the identification and classification of potato plant leaf abnormalityes. Further, early and late blight of potato, caused by Alternaria solani and Phytophthora infestans, respectively, require fundamentally different management strategies in agricultural practice. Early blight can be mitigated through timely curative measures when detected at an early stage, whereas late blight generally demands preventive intervention to avoid rapid disease spread and severe yield loss. In this context, automated disease recognition systems must go beyond mere classification accuracy and support disease-specific decision-making. Therefore, this research investigates the robustness of a DL-based object detection framework for the recognition of potato leaf diseases by addressing the limitations of existing approaches. Specifically, the proposed Inception-ResNetV2-based Faster R-CNN framework simultaneously localizes and classifies disease-affected regions, enabling early identification and reliable differentiation of potato leaf diseases. This capability supports timely and disease-specific crop management actions, thereby enhancing the practical applicability of automated disease detection systems in real-world agricultural environments. Specifically, an improved DL approach is presented by employing the Inception-ResNetV2 as the base network of the Faster-RCNN framework for the accurate localization and classification of numerous potato plant leaf ailments. At first, a set of dense keypoints is computed by the Inception-ResNetV2 approach, which is then passed and recognized by the 2-step detector of the Faster-RCNN model. A vast analysis of the proposed model is executed by using a standard dataset called the PlantVillage to show the effectiveness of this research. The contributions are listed as follows:

1. The proposed methodology employed an object detection approach called the Faster-RCNN, which integrates region proposal networks (RPNs) and CNNs, making it efficient in localizing disease-affected regions within potato plant leaves. This capability speeds up the detection process, enabling early intervention and treatment to mitigate crop losses.
2. The proposed methodology utilized the Inception-ResNetV2 architecture as the feature extractor, which is known for its deep and efficient keypoints computation, and significantly improves the accuracy of disease identification. This model’s multi-scale features and skip connections enable it to capture intricate details in potato plant leaves, enhancing the precision of disease classification.
3. The effective feature computation capability of the suggested approach enables it to handle diverse environmental conditions, including variations in lighting, weather, and plant health. This robustness ensures the model’s reliability across different agricultural settings, helping farmers identify diseases accurately under various circumstances.
4. An object detection approach is applied to both localize and classify the potato plant leaf diseases, which provides valuable data insights, including disease prevalence, distribution, and severity. These insights can be used for decision-making in agricultural practices, such as adjusting pesticide application, optimizing resource allocation, and implementing targeted disease management strategies, ultimately improving crop yield and reducing losses.
5. The proposed framework enables disease-specific agricultural decision-making by jointly localizing and classifying potato leaf diseases. This provides early-stage curative intervention for early blight (Alternaria solani) and preventive management planning for late blight (Phytophthora infestans), thereby directly linking DL-based detection with real-world crop protection schemes.
6. A huge experimental analysis is performed to verify the effectiveness of the suggested approach, which confirms that the proposed model is effective in detecting the early and late signs of potato plant leaf diseases under varying environmental and background settings.

The remaining article is organized as follows: Section 2 provides an in-depth discussion of existing works, while Section 3 elaborates on the suggested strategy. The experimental analysis is conducted in Section 4, and the conclusion, along with future directions, is addressed in Section 5.

## 2 Related work

This part of the paper presents a critical investigation of the technique presented for the identification of various potato leaf diseases.

Sinshaw et al (Sinshaw et al., 2022). presented a critical review of various models for classifying potato plant disease from the leaf samples. The approach has concluded that the DL approaches have been found more effective for the detection of potato diseases than the conventional ML methods. Mahum et al. (Mahum et al., 2023) utilized the concept of transfer learning to identify different types of diseases affecting potato plants. A DenseNet-201 approach utilized a comprehensive framework that covers the entire process from start to finish for dense feature engineering and categorization tasks. Further, to tackle the class imbalance in the data, the cross-entropy method was utilized. The approach shows effective results for recognizing different categories of potato leaf abnormalities; however, it lacks exact identification of the location of the infected regions. Amara et al. (Amara et al., 2023) showed a model by employing the idea of explainability to increase the credibility of attained results for classifying plant disease from images. A deep CNN approach was used in an end-to-end manner for computing the features and accomplishing the classification task. This approach (Amara et al., 2023) enhances the classification results; however, it lacks generalization ability. In (Tabbakh and Barpanda, 2023), a hybrid technique was suggested for categorizing various plant leaf diseases by utilizing the idea of transfer learning with a vision transformer. This technique adopted four key stages, where initially data samples were collected from the PlantVillage and wheat data samples. Next, data augmentation was performed to enhance the sample size. In the next stage, feature engineering was accomplished by using various DL-based approaches with the vision transformer approach. Finally, the categorization of the leaf diseases was performed in the classification layer by employing the computed key features. The approach attains the optimal results using the VGG19-based Vision transformer approach; however, it does face a drawback in terms of a high computational cost.

Zhao et al. (Zhao et al., 2022) proposed an approach in which an improved DL model was suggested by utilizing the Inception network with an attention mechanism approach for the classification of different types of diseases affecting potato leaves. Initially, a set of dense features was computed by using the suggested model, which was later recognized by the classification layer. This approach provides an efficient technique for potato plant disease classification; however, results need further improvements. In (Sachdeva et al., 2021), a model was introduced to identify plant leaf diseases with the support of DL. This work (Sachdeva et al., 2021) employed a Bayesian procedure-based residual framework for effective feature engineering processes, which were later classified by the classification layer. This approach (Sachdeva et al., 2021) shows better results in categorizing plant diseases of various types; however, it fails to process the distorted samples. Automated recognition of potato leaf blight diseases was proposed in (Chakraborty et al., 2022) from digital images. They implemented the four methods, VGG16, VGG19, ResNet50, and MobileNet on the PlantVillage database. Initially, the augmentation and preprocessing steps were performed for the preparation of data. The performance-wise top model is VGG16 with an accuracy of 92.69% after the parameter tweaking process. Moreover, the model performance over publicly available datasets is good; however, this model cannot be tested or validated on real-time samples. In (Hou et al., 2021), Hou et al. presented the graph cut segmentation approach for the identification of diseases of potato leaves from images. In the preprocessing phase, the authors utilized the Otsu thresholding for the extraction of seeds, and foreground and background were removed. After that, color and texture features were calculated, which were then classified through the KNN, SVM, ANN, and RF classifiers. SVM gave better results than the other classifiers with 97.4% accuracy. The method is unable to recognize the diseases in challenging scenarios like overlapping leaves and irregular illumination, etc. In (Anim-Ayeko et al., 2023), the authors proposed an automatic recognition approach for potato leaf diseases from images. The presented model, namely ResNet-19, is based on four convolutional, 4 residual layers, and one output layer. Performance was evaluated over the PlantVillage with 99.25% accuracy.

Rashid et al. (Rashid et al., 2021) suggested a method for categorizing diseases in potato plants. At first, the YOLO-V5 approach was utilized for the localization of regions. Subsequently, CNN was suggested to capture the visual features of the provided samples and carry out the classification task. The study in (Rashid et al., 2021) performs well in recognizing the diseased samples of potato plants from healthy ones; however, it fails to tackle the infected areas of small size. Ullah et al. (Ullah et al., 2023) planned the DeepPlantNet to categorize numerous types of plant leaf diseases. The framework contained 28 layers, of which 25 layers were dedicated to computing features, while the remaining 3 were fully connected layers. This model (Ullah et al., 2023) shows effective results in accomplishing plant ailments recognition; however, it fails to exactly locate the region of interest. Another similar approach was proposed in (Khalifa et al., 2021), which was focused on recognizing the healthy samples of potato plants from the diseased images. Initially, an augmentation was utilized to improve the size and diversity of the dataset. After this, a DL model was suggested comprising 14 layers for feature engineering and classification tasks. Afzaal et al (Afzaal et al., 2021). employed the idea of transfer learning to identify diseases in potato leaves. To accomplish this, the approach performed an analysis of 3 DL frameworks named GoogleNet, VGGNet, and EfficientNet, and found that EfficientNet performs well in comparison to other models. Another approach using the idea of a pre-trained model was suggested in (Chen et al., 2023), where the light architecture approach called MobileNet V2 was investigated to classify the potato plant leaf diseases. This model (Chen et al., 2023) provides an efficient solution to distinguish between healthy and diseased leaf samples; however, classification performance needs improvement. Reddy et al. (Sai Reddy and Neeraja, 2022) suggested an automated model to perform the classification of numerous plant leaf ailments. The method initially employed DenseNet for the classification of samples into their respective classes. After this, a DL model was used to accomplish the semantic segmentation of the diseased regions of plant leaves. The approach (Sai Reddy and Neeraja, 2022) shows effective results for plant leaf disease classification, though the model needs testing on large datasets. Arshaghi et al. (Arshaghi et al., 2023) also investigated a CNN approach in recognizing the healthy samples of potatoes from diseased ones. The work (Arshaghi et al., 2023) performs well in accomplishing the classification task; however, it is unable to tackle the distorted samples. Another ML-based approach was presented in (Singh and Kaur, 2021) for classifying leaf diseases. Primarily, the K-means approach was used to locate the region of focus. Next, the gray-level co-occurrence matrix was used for the feature engineering process, while the SVM was adopted to perform the classification task. This work (Singh and Kaur, 2021) shows efficient solutions to potato plant leaf diseases; however, classification results need improvements. Saha et al. (Saha et al., 2025) suggested a custom lightweight CNN model for potato leaf disease detection, trained on the PlantVillage dataset. The approach integrates CLAHE-based image enhancement during preprocessing, enabling the model to achieve a high accuracy of 99.30%; however, the work is ineffective in dealing with distorted images and locating the exact diseased portion. Kumari et al. (Kumari et al., 2025) proposed an ML approach for potato plant leaf classification, in which approaches like Hough Transform (HT) and Discrete Wavelet Transform (DWT) were used across different color spaces. The computed keypoints were classified using multiple ML predictors, with logistic regression (LR) achieving the highest accuracy of 99% in the YCbCr color space. The work lacks to locate the exact location of the diseased region. Further, the work in (Reis and Turk, 2024) proposed a DL model that combined depthwise separable convolutions with a multi-head attention mechanism to classify potato leaf diseases accurately. The model was further integrated with machine learning classifiers such as SVM and ensemble methods, achieving a highest accuracy of 99.33%. This work emphasizes disease classification but does not address disease localization.

The performed analysis shows that huge efforts have been put in by the researchers for the timely and accurate recognition of early and adverse types of potato plant leaf diseases; however, existing works face several challenges. First, the diversity of disease symptoms and the potential for overlap between diseases can make accurate classification a complex task. Second, the limited availability of diverse and well-annotated datasets hinders the development and evaluation of robust models. Additionally, distinguishing between visually similar diseases poses a significant challenge. The need for an effective solution that can adapt to varying environmental conditions and accommodate different potato varieties further complicates the task. Addressing these challenges is essential for advancing the field of leaf disease identification and supporting effective disease management in agriculture.

## 3 Methodology: PotatoGuardNet

**Figure 1:**
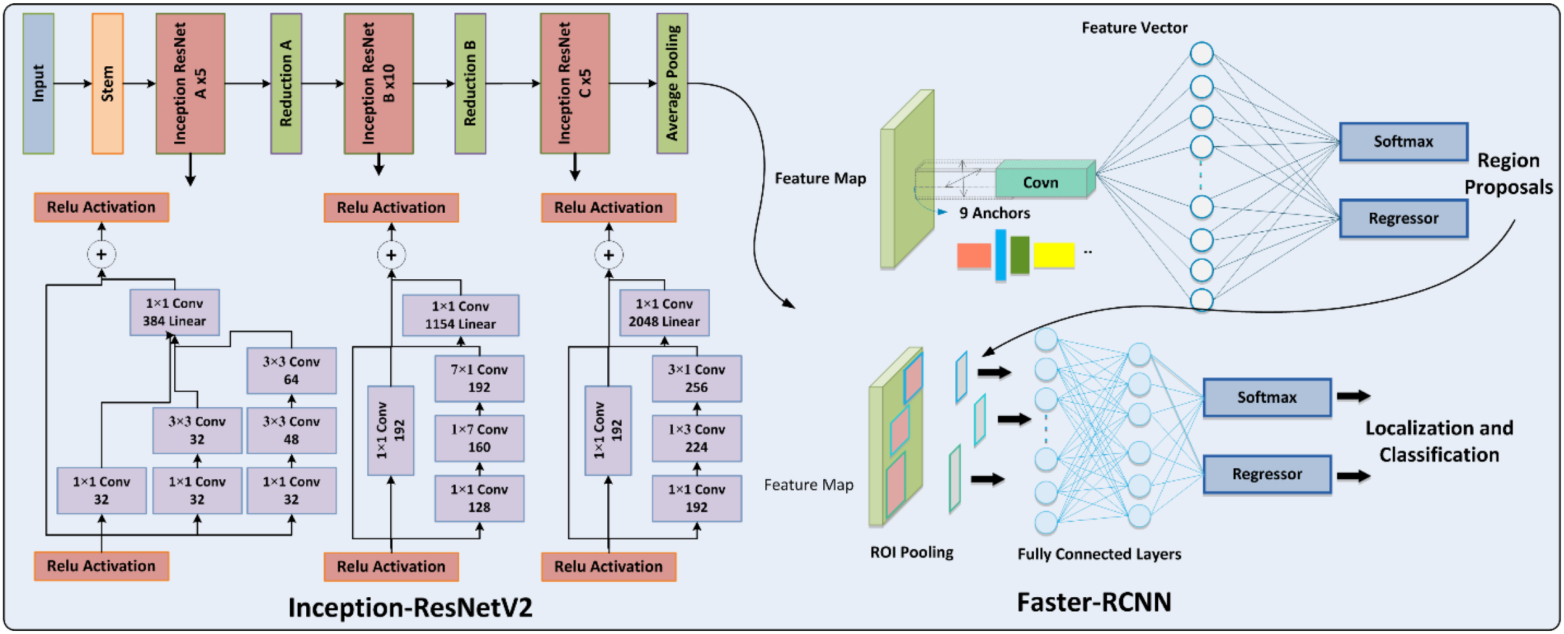
PotatoGuardNetFramework.

The PotatoGuardNet is established on the custom Faster RCNN approach, which is efficiently customized for potato detection. Choosing the feature extraction method is a crucial step in the field of computer vision. Specifically, the InceptionResNet-V2 approach is employed as the base network to capture the visual attributes of the samples, which are subsequently identified and categorized by the 2-stage detector within the Faster-RCNN. The suggested model is trained and validated on leaf samples. The complete architecture of the custom model is illustrated in **Figure 1**, providing evidence of superior recognition of potato leaf diseases.

### 3.1 Feature extraction using Faster-RCNN

This section demonstrates the proposed methodology with hyperparameter details. To identify potato leaf diseases from images, the proposed mechanism employed the Faster-RCNN (Ren et al., 2016; Albahli et al., 2021) methodology that can recognize objects precisely. The proposed technique improved this method by using the Inception-ResNetV2 approach, which can effectively determine the features. In the field of agriculture, it is necessary to calculate the effective and reliable features to recognize the disease areas in the images. Further, it’s very important to avoid illogical behavior because of larger feature sets, and also to avoid missing the important features due to smaller sets. It can be asserted that feature extraction plays a crucial role in identifying disease areas from colored images.

In the Faster-RCNN approach, there are convolving filters used for the structure analysis of images to obtain the required features. The main aim of choosing this technique is the RPN module, which is a better strategy for feature extraction as compared to the RCNN model. Furthermore, RCNN and Fast-RCNN are dependent on a selective search approach-based and hand-crafted strategy. As these methods are manual, so have many issues like being time-consuming, computationally complex, and error-prone. Furthermore, this methodology is divided into two main modules: RPN and Fast-RCNN. The RPN is utilized for the generation of object proposals, which is an automatic process, and then passed as input to the next module. In the Fast-RCNN module, generated proposals are then refined through the convolutional layer (CL). After that, input is transferred once using CNN to create and improve the object proposals.

### 3.2 Inception-ResNetV2

The Inception network architecture, as described in reference (Vedaldi and Zisserman, 2016), leveraging multiple convolution kernels of variable sizes, is employed to improve the adaptability of the network and capture a broader spectrum of features across different scales. Simultaneously, it effectively reduces the model’s parameters by adopting the NIN (Paul et al., 2020) technique, following the principle of minimizing convolution kernels while preserving feature representation, thus reducing overall model complexity. The architecture of the residual network enables direct signal propagation between different units and layers in both forward and backward directions, significantly expediting the process of training.

Within the inception-resnetv2 architecture, the residual convolution network allows for flexibility in the number of feature maps in different input layers (‘*li*’). Due to this variability, situations may arise where the quantity of feature maps in a particular layer differs from those in another. To address these discrepancies and ensure smooth transitions between layers, 1 × 1 convolutions are employed strategically. These convolutions serve to adjust the dimensionality, either increasing or decreasing the number of feature maps, facilitating the network’s ability to maintain information flow and hierarchical representations across layers effectively. The residual procedure is calculated using Equationa 1–3:

(1) $$F(l_{i})=w\times l_{i}+\alpha$$

(2) $$m_{i}=R(F)+h(l_{i})$$

(3) $$l_{i+1}=R(m_{i})$$

Where, *l_i_* = input, *m_i_* = Sum, *w*= weight, α=offset, *R*= Relu as calculated in Equation 4.

(4) $$R(l_{i})=\max(0,l)$$

For *l* > 0, its output is 1; otherwise, it has an output of 0. During forward computation, the input value is l, and the threshold is set to 0 to get the outcome. In the opposite case, the gradient is either 0 or 1. When compared to Tanh and Sigmoid activation functions, the ReLU function is simpler to compute and has a shorter gradient decrease. This property makes it advantageous for deepening networks.

The introduction of a residual learning unit aimed to address the issue of gradient vanishing during the training of the Inception network model. Simultaneously, when the performance touches a reliable saturation level, the residual layer can map inputs equally, which accelerates training and facilitates convergence. *Li* and *Ln* represent the input of the *i^th^* and *n^th^* units, respectively. Equation 5 describes the acquisition of properties from layer *i* to *n*. Certainly, the gradient will never approach 0 regardless of how deep the layers are.

(5) $$\frac{\partial L_{n}}{\partial L_{i}}=\frac{\partial L_{i}+F(L_{i},\omega_{i},\alpha_{i})}{\partial L_{i}}=1+\frac{\partial F(L_{n},\omega_{n},\alpha_{n})}{\partial L_{n}}$$

This work utilized a three-layered residual model, which employed a 1 × 1 convolution for dimension reduction, followed by a 3 × 3 convolution. Notably, the three-layer residual network units exhibit a remarkable reduction in the number of network parameters, approximately 17.35 times less than their two-layer counterparts. However, the original Inception module (IM) has limited efficacy in enhancing network performance, while its enhanced versions can be overly complex, resulting in a high parameter and computation load, which often leads to overfitting. The network might possess adequate width but lacks depth, causing an imbalance that hinders parameter operations’ efficiency.

On the other hand, the ResNet module, while deepening the network and improving classification accuracy, rapidly increases the number of parameters and computations. This is faster than the Inception; however, it suffers from the limitation of a relatively narrow network width, leading to less diverse feature extraction than the IM. When the Residual module becomes overly complex, the benefits of skip connections may be overshadowed by the critical increase in constraints and estimates, potentially causing training interruptions or gradient explosions.

**Figure 2:**
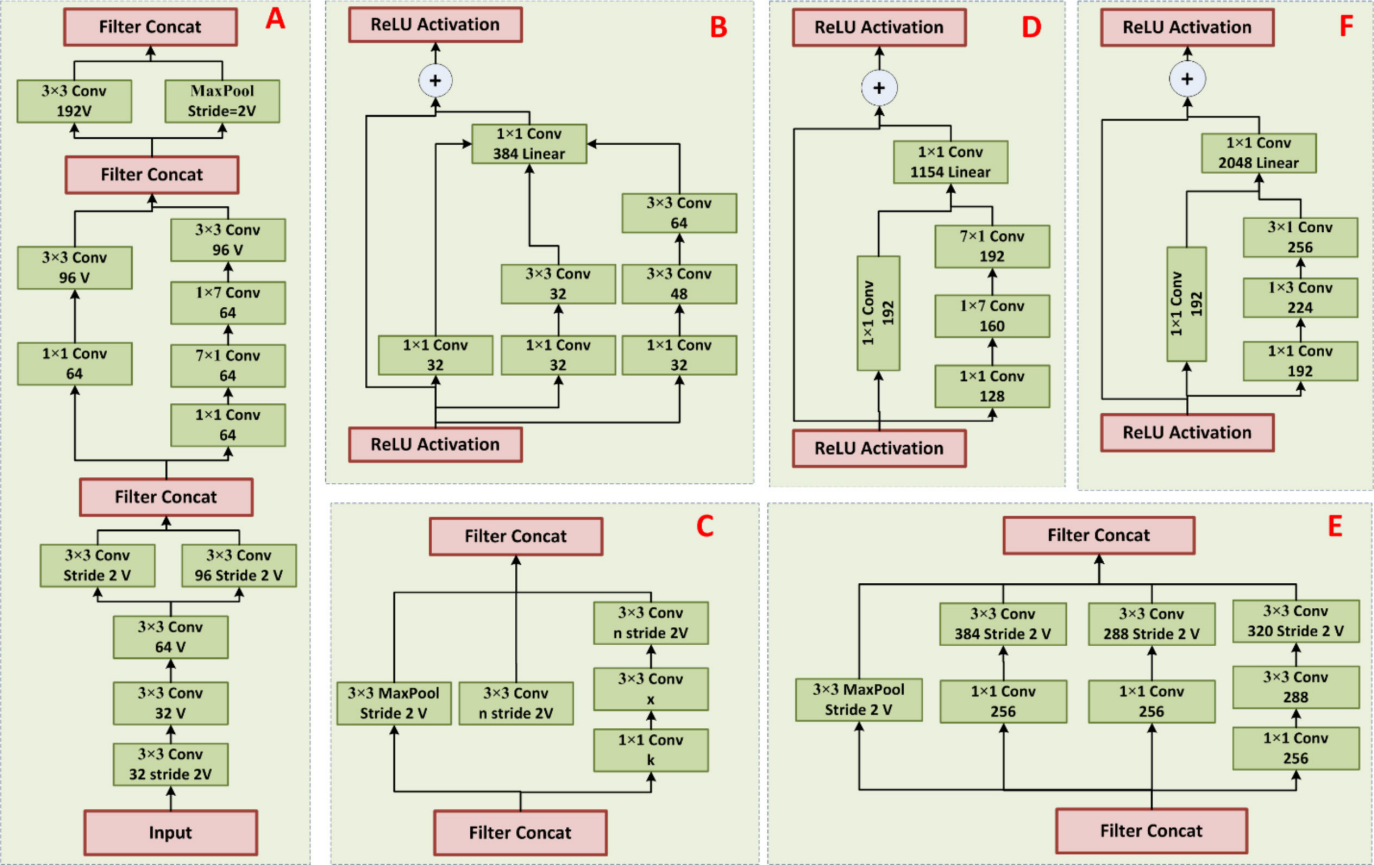
**(A–F)** Modules of InceptionResNet.

To tackle the above shortcomings, this research presented the Inception-Resnet-v2, which is able to enhance detection accuracy while reducing the computational load. The presented model (as shown in **Figure 2**) has three major components, one of which is the “stem,” which has deep CLs responsible for preprocessing the original data. The stem encompasses 9 convolutional and 2 max-pooling layers. The next component is composed of different modules; the InceptionResNet-A module (can be seen in **Figure 2A**), containing 3 × 3 kernels in the IM. In **Figure 2C**, the Inception-ResNet-B module is depicted, featuring an asymmetric filter combination with one 1 × 7 and one 7 × 1 filter in the IM. **Figure 2E** highlights the Inception-ResNet-C module, utilizing a smaller and asymmetric filter combination of one 1 × 3 filter and one 3 × 1 filter. Moreover, 1 × 1 convolutions are incorporated before the larger filters in these modules, enhancing the diversification of filter patterns through asymmetric convolution splitting. To address the dimensionality decrease affected by the Inception block, **Figures 2B, D** introduce reductions in A and B, aiming to increase the dimension within the network.

In the computer vision field and real-world applications, two primary challenges persist: accurately determining the precise locations of multiple objects within an image and correctly identifying the class to which each detected object belongs. When it comes to detecting and recognizing potato leaf diseases in images, Faster Regional Convolutional Neural Network (R-CNN) addresses these challenges effectively by employing the Fast R-CNN with RPN, which leverages information regarding object characteristics such as size, color, and more to detect both the class and location of objects, leading to enhanced performance. Additionally, it reduces the overall computational burden, yielding favorable results. This approach encompasses the following key steps:

### 3.3 Convolution layers

Inception-ResNet-V2 is composed of a series of consecutive residual blocks, with each block containing CLs and identity shortcuts. The CLs within the residual blocks play a crucial role in extracting features from the input image. Typically, each residual block consists of multiple CLs, often with a size of 3×3. Following these CLs, batch normalization, and the ReLU are applied to introduce non-linearity.

Let the input potato leaf image be denoted using Equation 6.

(6) $$I\;\in \;R^{H\times W\times 3}$$

When resized to 299×299×3, the feature extractor computes a deep feature map using **Equation 7**.

(7) $$F\;=\;\Phi \left(I;\;\theta_{b}\right)$$

- Where Φ(·) represents the Inception-ResNetV2 backbone,
-
$\theta_{b}$ denotes the learned parameters,
-
$F\;\in \;R^{H^{\prime }\times W^{\prime }\times C}$ is the extracted multi-scale deep feature representation obtained from the Inception-ResNetV2 backbone, where *H′* and *W′* denote the spatial dimensions after convolution and *C* represents the number of feature channels. The residual learning mechanism is expressed using Equation 8.

(8) y=F(x)+x 

Where F(·) denotes the Inception block transformation, and *x* is the input feature map. The resulting feature map is subsequently passed through the RPN and related interconnected layers.

### 3.4 RPN

The primary role of the RPN is to suggest potential areas within an image that might contain objects. These proposed regions are subsequently used as input for the subsequent stages of object recognition. The RPN accomplishes this by scanning the convolutional feature map of the input image with a small sliding window, typically measuring 3×3. At each window position, the RPN makes predictions regarding the presence of an object within the window and adjusts to better align the window with the object, enhancing accuracy.

### 3.5 Anchor boxes

In the RPN, a collection of pre-defined anchor boxes with varying sizes and aspect ratios is employed. These boxes are positioned at the center of each sliding window on the feature map. The RPN’s task is to determine whether each anchor box contains an object, and if it does, to predict the necessary adjustments (refinement offsets) to accurately align the anchor box with the object’s location.

Here, Anchors of different scales and aspect ratios are generatedat each spatial location of *F* using Equation 9.

(9) $$A\;=\;\left\{a_{i}\right\},\;i\;=\;1,\;2,\ldots ,\;N$$

Each anchor *A* is classified as foreground or background and regressed to a refined bounding box. The RPN loss is defined using Equation 10.

(10) $$L_{RPN}=\;\left(\frac{1}{N_{cls}}\right)\;\Sigma \;L_{cls}\left(p_{i},\;p_{i}^{*}\right)+\text{ λ }\left(\frac{1}{{\text{N}}_{\text{reg}}}\right){\text{ Σ p}}_{\text{i}}^{*}L_{reg}\left(t_{i},\;t_{i}^{*}\right)$$

- where $p_{i}$ is the predicted objectness score,
-
${\text{p}}_{\text{i}}^{*}$ is the ground truth label,
-
$t_{i}$ and $t_{i}^{*}$ are predicted and ground-truth bounding box parameters.

### 3.6 Classification network

The role of the classification sub-network is to determine whether each anchor box contains an object or represents a background region. It achieves this by predicting an objectness score for each anchor box, which signifies the probability of the box containing an object. The classification network employs a cross-entropy loss function to train and optimize these objectness scores.

### 3.7 Regression network

The regression sub-network is responsible for estimating the refinement offsets for anchor boxes that have been classified as containing objects. These offsets are applied to modify the positions and sizes of the anchor boxes, improving their alignment with the actual locations and shapes of the objects. To train and optimize the refinement offsets, the regression sub-network employs the smooth L1 loss. Bounding box refinement is computed using Equation 11.

(11) $$t_{x}\;=\frac{\left(x\;-\;x_{a}\right)}{\;w_{a}}\text{, }\;t_{y}\;=\frac{\left(y\;-\;y_{a}\right)}{h_{a}}\text{, }t_{w}\;=\log\left(\frac{w}{w_{a}}\right)\text{, }t_{h}\;=\;\text{log}\left(\frac{h}{h_{a}}\right)$$

where

- *x, y, w, h* denote predicted box coordinates,
-
$x_{a}$, $y_{a}$, $w_{a}$, and $h_{a}$ denote anchor box parameters.

### 3.8 Non-maximum suppression

Once the objectness scores and refined bounding box coordinates have been acquired for all anchor boxes, a crucial step is to apply NMS, which is used to eliminate duplicate and highly overlapping proposals. It ensures that only the most confident and non-overlapping proposals are retained, reducing redundancy and computational load in the subsequent stages of the object detection process. For more technical details, the readers can refer to (Ren et al., 2016).

### 3.9 Overall multi-task loss function

The total loss of the proposed work is defined using Equation 12.

(12) $$L=L_{cls}\;+\;L_{reg}$$

- where $L_{cls}\;$ is the classification loss (cross-entropy),
-
$L_{reg}$ is the Smooth-L1 bounding box regression loss.

## 4 Results

In this part of the paper, a vast analysis of the suggested approach is conducted to demonstrate the model’s effectiveness in recognizing several forms of potato leaf ailments. For this reason, various standard performance measuring metrics are utilized to test the approach on a huge data sample repository. A comprehensive explanation of the measures utilized, the dataset, etc., can be found in the succeeding units. Further, the presented framework was implemented using Python and executed on an Nvidia GTX1070 GPU-based system. Model training was performed using the Adam optimizer with an initial learning rate of 0.0001 and a mini-batch size of 16. The network was trained for 50 epochs, and categorical cross-entropy loss was employed for classification, along with the standard bounding-box regression loss used in Faster R-CNN. All input images were resized to 299 × 299 pixels to satisfy the input requirements of the Inception-ResNetV2 backbone. Training and inference were conducted on a single GPU without distributed or multi-GPU scaling.

### 4.1 Evaluation parameters

To assess the performance of the introduced approach, respective standard evaluators are chosen, like precision value, recall measure, accuracy metric, F1-measure, intersection over union (IoU), and mAP. The mathematical representation of the mAP is provided in **Equation 13**, where *s* designates the analyzed sample, while *S* shows the total data samples.

(13) $$mAP:=\sum_{j=1}^{S}AP(s_{j})/S$$

**Figure 3:**
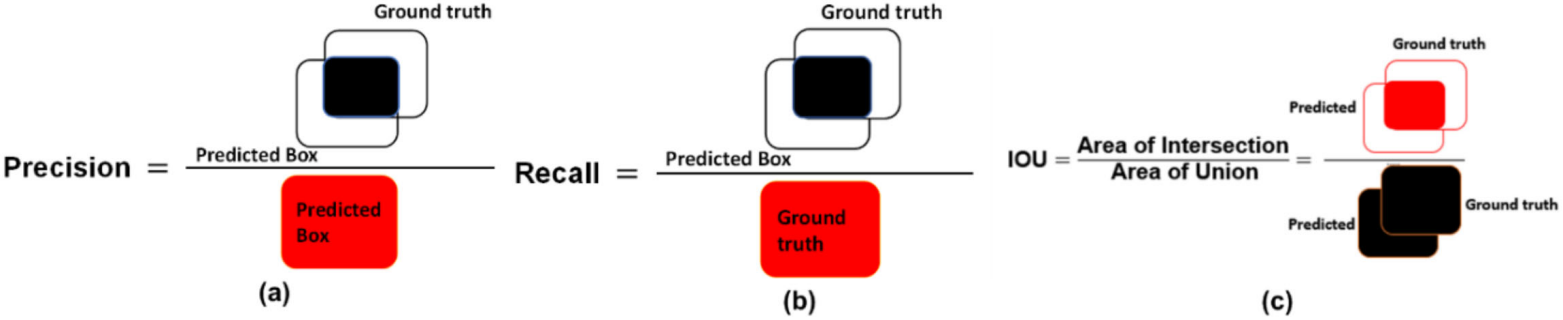
Pictorial illustration of **(a)** Precision, **(b)** Recall, and **(c)** IOU evaluators.

The visual representation of the precision, IOU, and recall metrics is given in **Figure 3**.

### 4.2 Dataset

**Figure 4:**
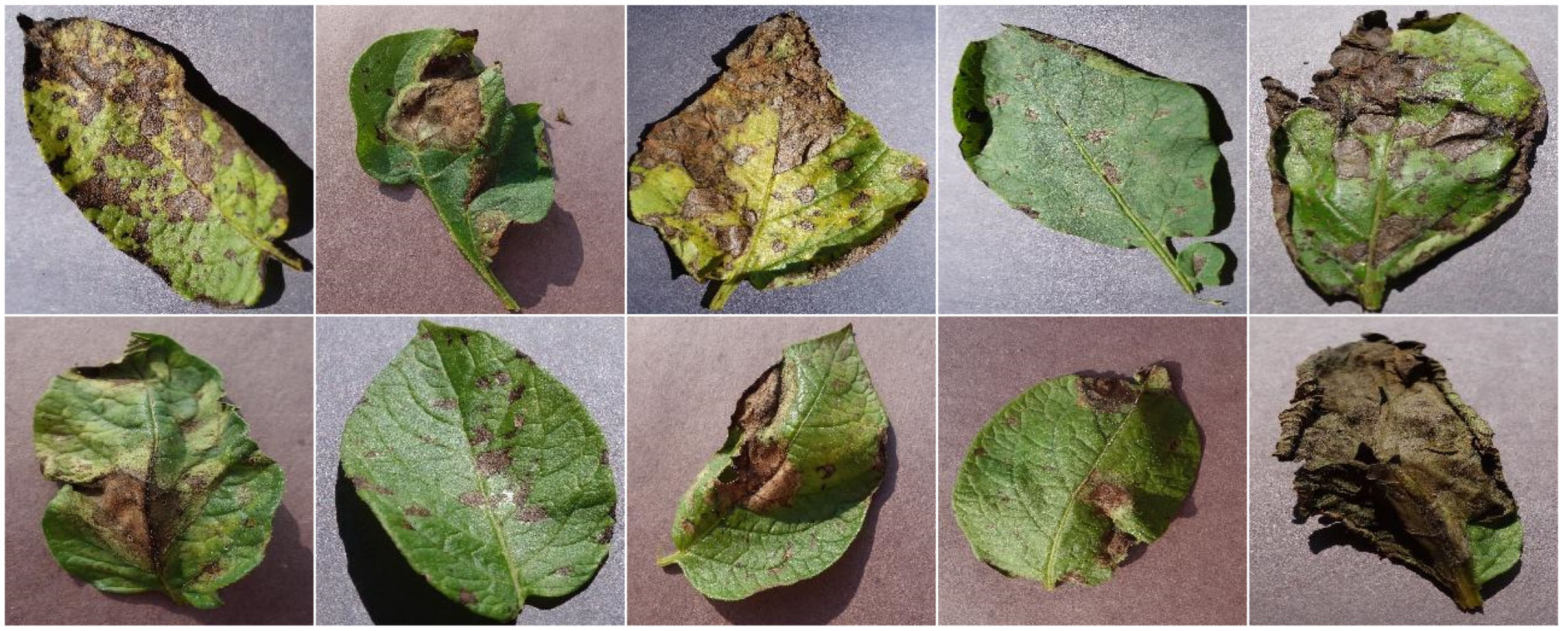
A few examples from the utilized data sample.

To tune and test the proposed work, a standard and online available data sample called the PlantVillage (Hughes and Salathé, 2015) is utilized, which is a thorough group of high-quality images of leaves affected by various diseases. It serves as a valuable resource for training and evaluating various approaches for plant disease identification and classification. The data sample presents a vast assemblage of plant leaf samples with 54,306 examples from fourteen different categories. Each image is provided in JPEG format with varying native resolutions ranging from 256×256 to over 1024×1024 pixels. For this work, we specifically used the potato subset, which includes three major classes: Healthy, Early Blight, and Late Blight, with a total of approximately 4,500 annotated leaf images. All samples were standardized to 299 × 299 × 3 to meet the input requirements of the Inception-ResNetV2 feature extractor. These classes contain images showcasing the visual symptoms and characteristics of each potato disease, aiding researchers and agricultural professionals in developing and fine-tuning models for the early detection and recognition of potato plant diseases. The data sample exhibits significant data variability, incorporating images from diverse sources and locations with varying lighting, backgrounds, and image quality, making it challenging to build models that can be generalized effectively. Such a dataset’s diversity and focus on potato-related classes make it an essential choice for addressing the specific challenges of potato cultivation and disease control. **Figure 4** shows several sample images.

### 4.3 Localization results

**Figure 5:**
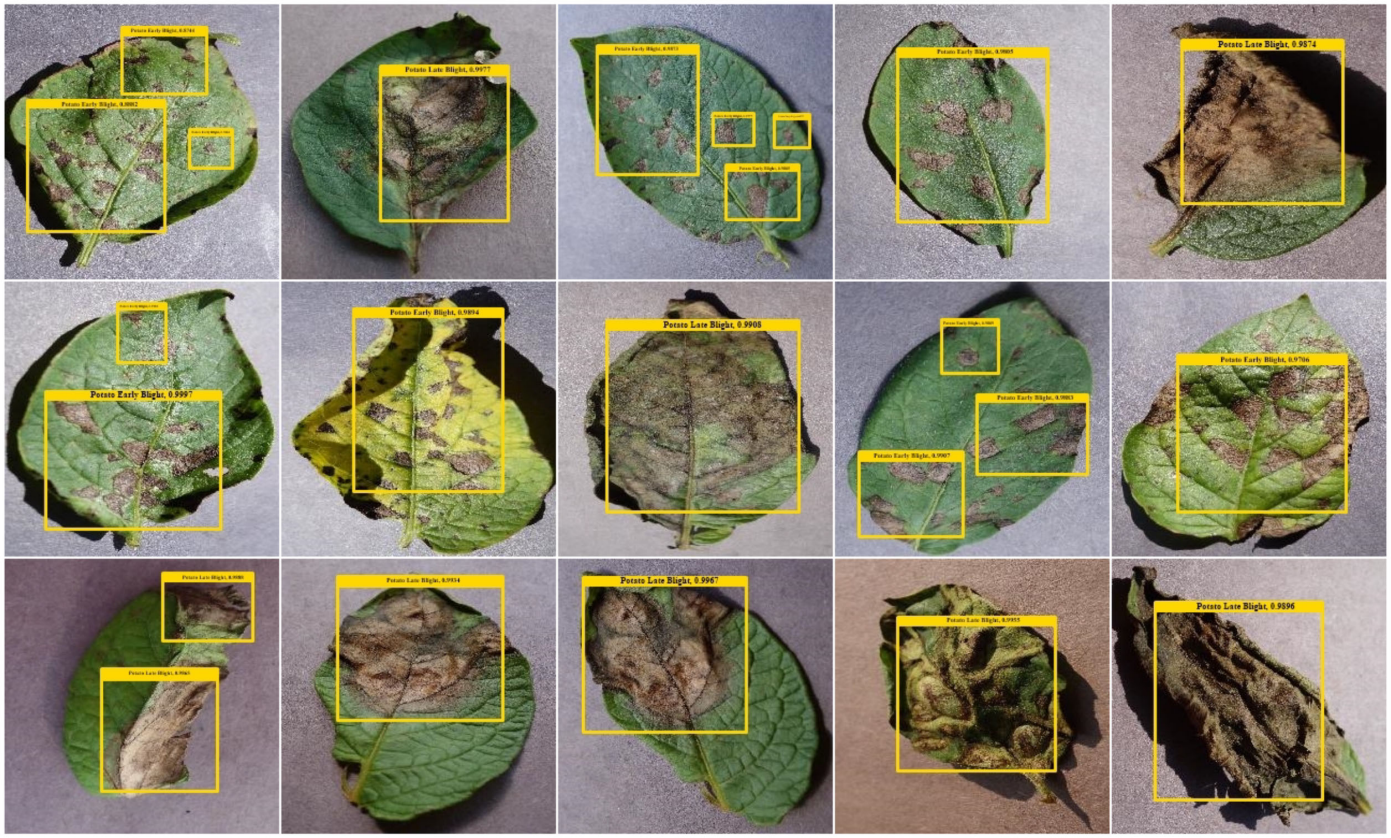
Localized samples by the proposed work.

First, the localization results of the proposed technique are utilized, as in the context of plant leaf disease recognition, computing localization results using metrics like mAP and IoU is highly significant. These metrics assess the precision and accuracy of disease localization within plant leaves. The mAP metric presents a thorough assessment of the model’s ability to correctly categorize and localize disease-affected areas of potato plants while considering both false positives and false negatives. This holistic assessment is critical for ensuring the model’s reliability in applications like precision agriculture, where accurate disease detection can significantly impact crop health. Additionally, the IoU metric precisely measures the spatial overlap between the predicted disease regions and the ground truth annotations, ensuring that the model’s localizations are in close alignment with actual disease areas. By utilizing these metrics, the discussion concerning the effectiveness and trustworthiness of the suggested approach is conducted in this part of the paper. A mAP score of 0.9556 is attained, along with an IOU of 0.9504, which clearly depicts the effectiveness of the proposed technique and shows that it supports early and accurate disease diagnosis in agriculture, which can contribute to improved crop management. Further, the visual results are exhibited in **Figure 5**, which shows the proposed method is proficient in recognizing potato plant diseases in both early and severe stages under the existence of huge alterations, which proves the robustness of the proposed model.

### 4.4 Heatmaps analysis

**Figure 6:**
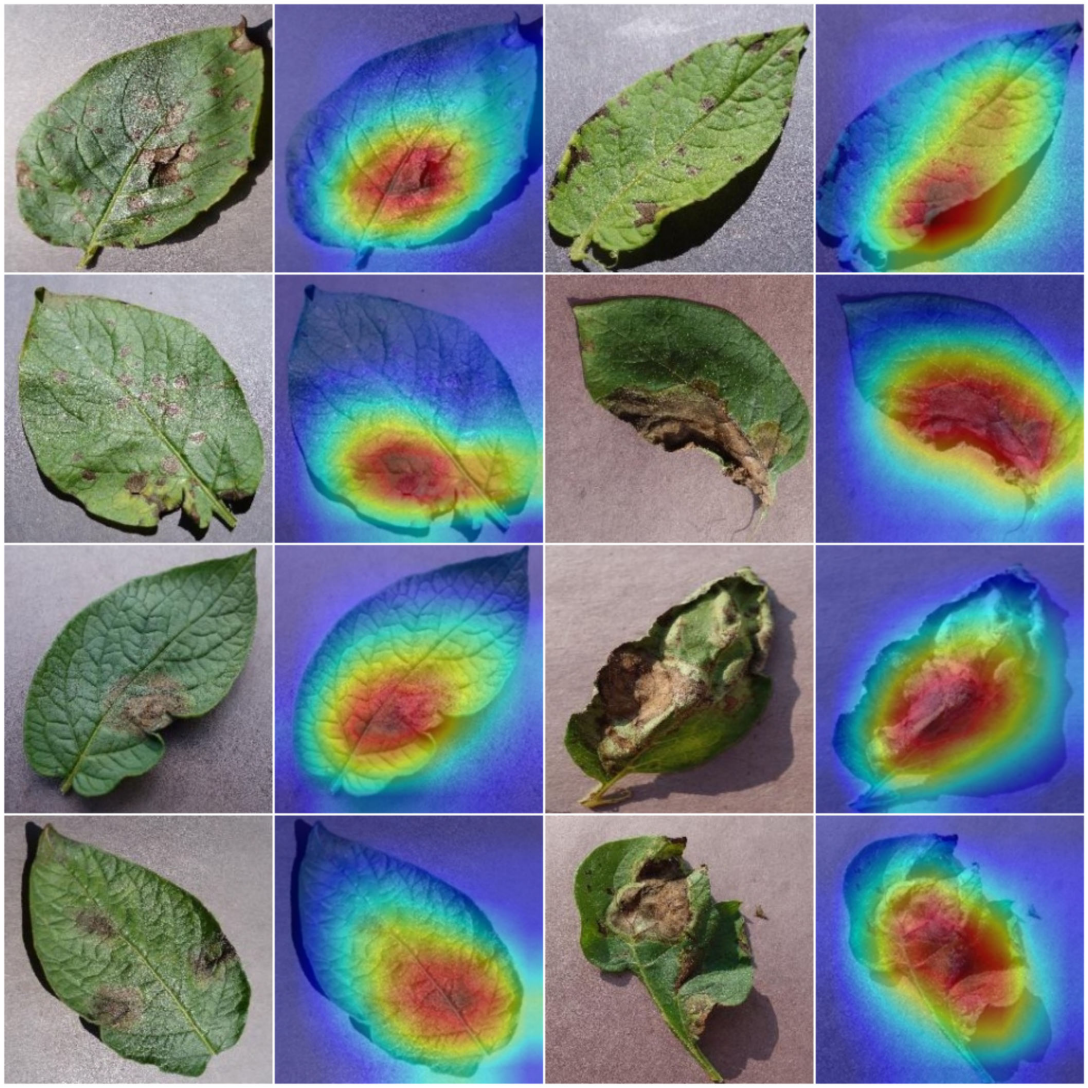
Heatmaps generated by the proposed approach.

To further show the robustness of the approach, the demonstration of heatmaps as generating heatmaps in the context of plant leaf disease recognition holds great significance, as it provides a visual representation of the model’s disease localization. Heatmaps offer insights into where the model identifies disease symptoms on the leaf, helping researchers and farmers pinpoint affected areas with precision. This visualization aids in understanding the model’s decision-making process and can be used to validate the accuracy of disease predictions. Heatmaps also serve as an educational tool, enabling researchers and domain experts to grasp the severity and extent of disease infections. Furthermore, they empower early intervention by allowing farmers to take targeted measures to treat or remove affected portions, ultimately improving crop health and minimizing losses. Collectively, heatmaps bridge the gap between technical model outputs and practical on-field applications and serve as an important tool to validate the effectiveness of an approach. For this reason, the Grad-Cam tool is utilized to attain the heatmaps against the last convolution layer of the model to highlight the regions in an image that contribute the most to a framework’s decision for a specific class. The obtained results are provided in **Figure 6**. The highlighted red and yellow areas correspond to the most influential regions contributing to the classification decision, typically aligning with symptomatic patterns such as lesions, spots, discoloration, and necrotic tissues. In contrast, blue regions represent areas with minimal impact on the model’s prediction. These visualizations confirm that the model correctly attends to disease-specific features rather than irrelevant background regions, demonstrating both interpretability and reliability of the proposed framework. The results prove that the proposed model has taken the correct regions to accomplish the recognition task due to its high recall rate.

### 4.5 Model performance evaluation

In this section, a thorough examination of the proposed framework is discussed in detail by explaining the results both in the aspect of individual class, and on the dataset with the assistance of measures like precision, recall, accuracy, error rate, and F1 measures.

**Figure 7:**
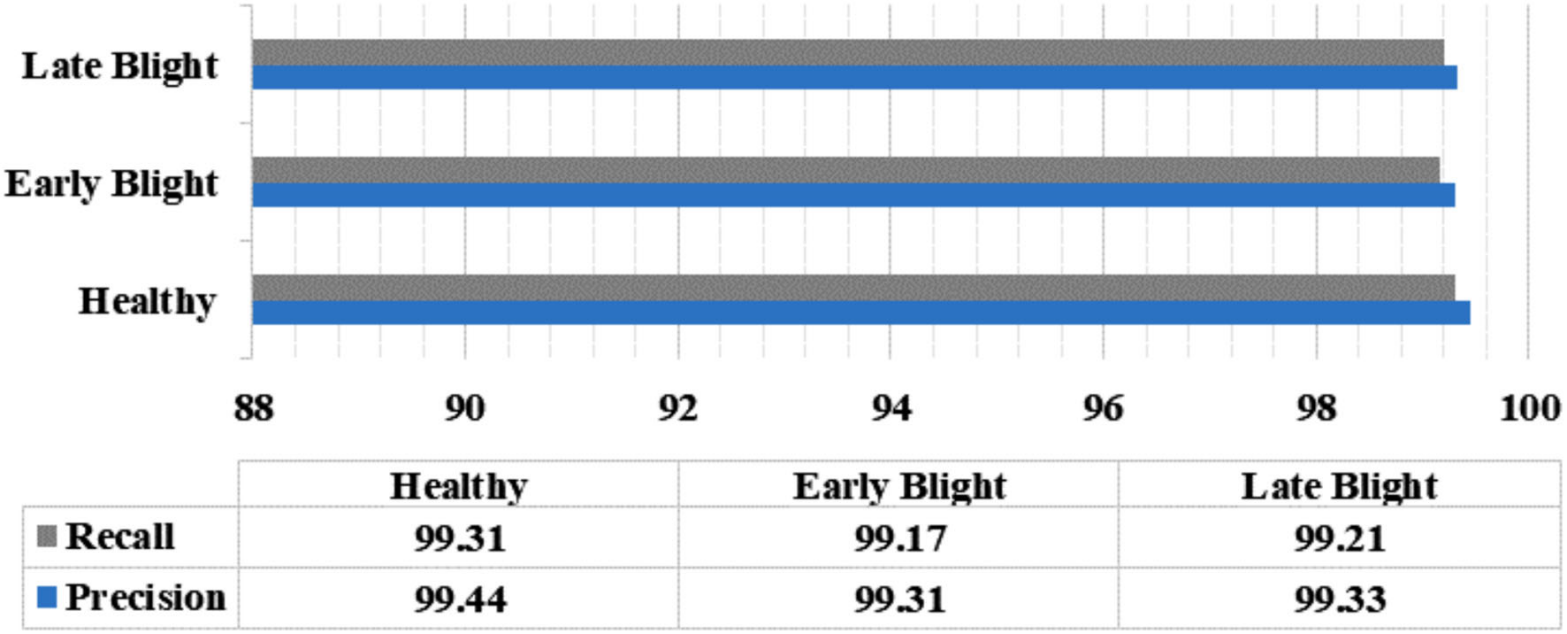
Precision and recall values for all categories.

First, the class-wise numeric score of the model is explained by using several evaluation measures that offer a comprehensive estimation of the model’s performance, not just in identifying diseases but also in distinguishing among various classes, which is crucial in applications like plant leaf disease recognition. These parameters give insight into the ability of the technique to correctly classify different diseases, quantify the extent of false positives and false negatives, and ultimately aid in assessing its reliability and effectiveness. Such a detailed analysis not only helps researchers fine-tune the model for better overall performance but also supports practical decision-making in agriculture, enabling more accurate and targeted disease management strategies for each specific class of plant diseases. Initially, outcomes are elaborate with respect to group-wise attained precision and recall values, as precision measures the accuracy of positive predictions within each class, providing insights into how often the model correctly identifies specific diseases. While recall, on the other hand, quantifies the model’s ability to capture all instances of a particular disease, minimizing false negatives. The attained comparison is provided in **Figure 7**, from where it can be witnessed that the model shows effective scores for classifying all groups of potato plant leaves. The proposed method has attained the class-wise precision scores of 99.44%, 99.31%, and 99.33% for healthy, early, and late blight potato plant leaf groups, which are 99.31%, 99.17%, and 99.21% in terms of recall measure, which is proving the high classification performance of the proposed methodology.

**Figure 8:**
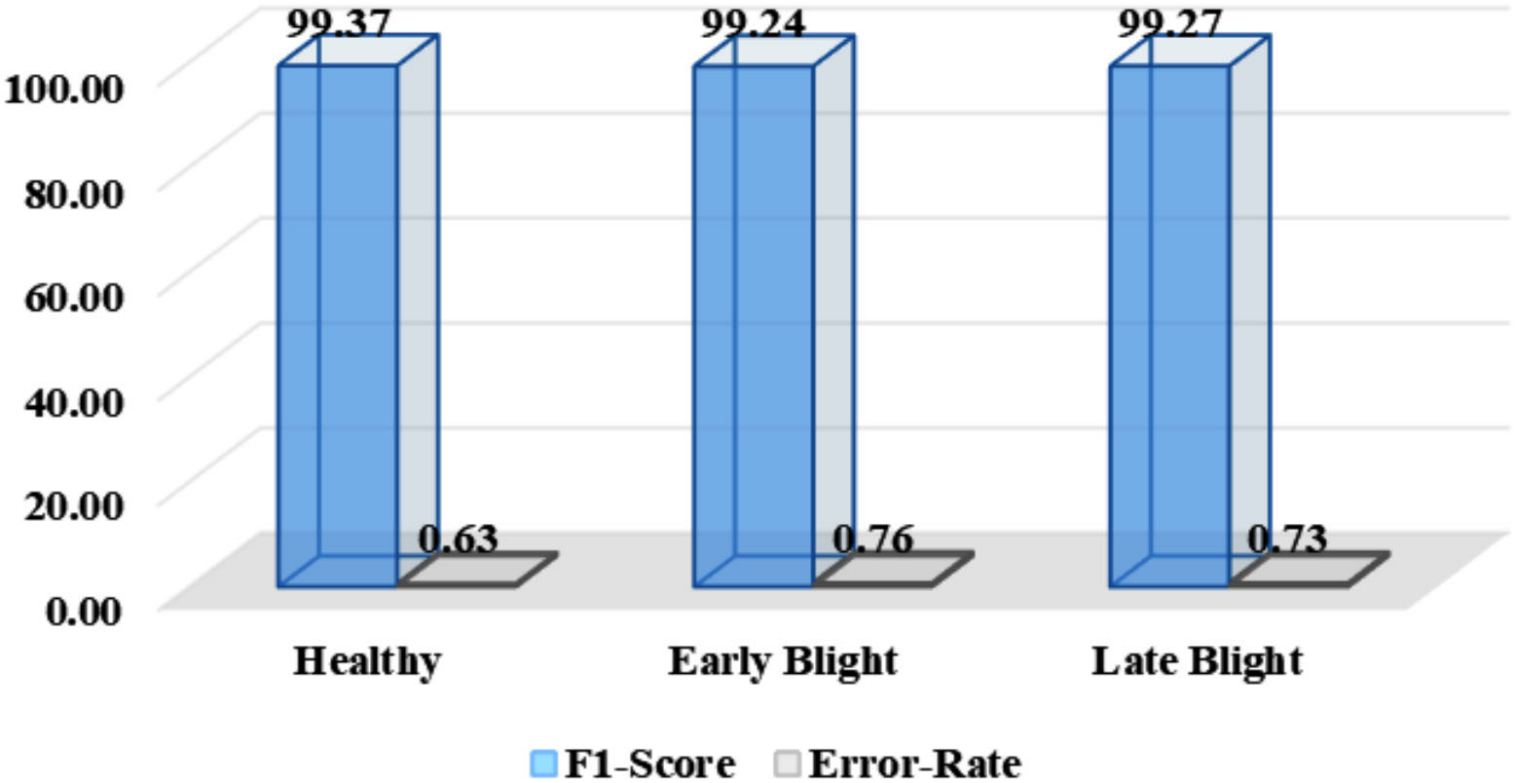
Group-wise F1 and error values attained by the proposed model.

Next, the discussion covers the F1-score and error rates for all categories of potato leaves. The major reason to compute the F1-score is that it harmonizes precision and recall by providing a unified metric that encapsulates the model’s capability to achieve both high accuracy and exhaustive disease detection within each class. Further, class-wise error rates assist in seeing the struggling areas of a model. The attained values for all three classes are given in **Figure 8**, from where it is quite evident that the proposed method has obtained high F1 values for all groups of potato leaves with small error rates against all groups. Noticeably, this research work accomplished an error rate of 0.71% indicating high recognition power of the proposed model.

**Figure 9:**
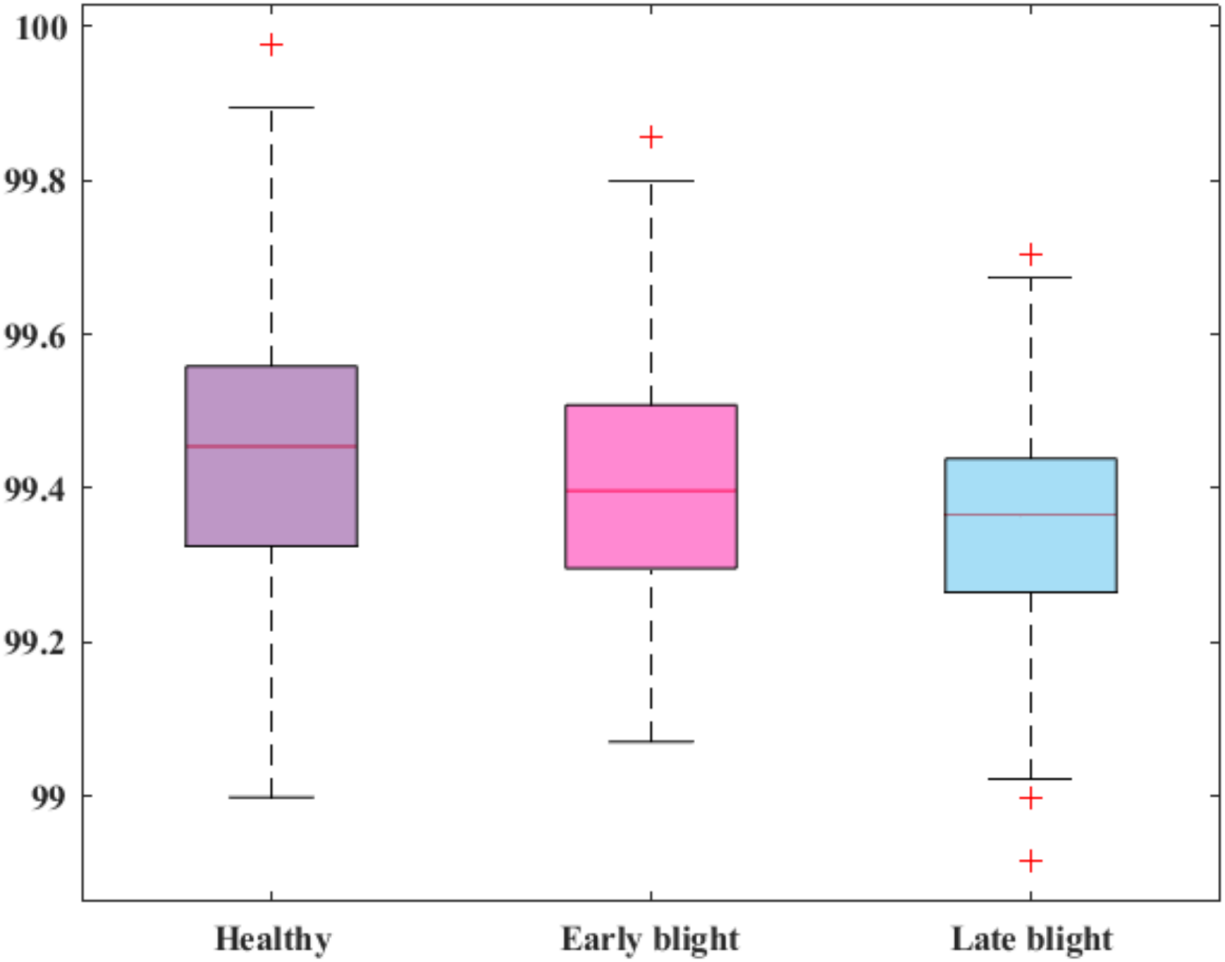
Accuracy scores of the model for all classes of potato leaves.

Next, the accuracy scores of all categories of potato leaf diseases are reported, as computing class-wise accuracy values is significant because such computation agrees with a detailed assessment of model performance across individual disease categories in plant leaf disease recognition. Unlike overall accuracy, which provides a single number for the entire dataset, class-wise accuracy reveals how well the model is performing for each specific class. The accomplished outcomes are provided in **Figure 9**, and it can be concluded that satisfactory performance was observed across the entire categories.

**Figure 10:**
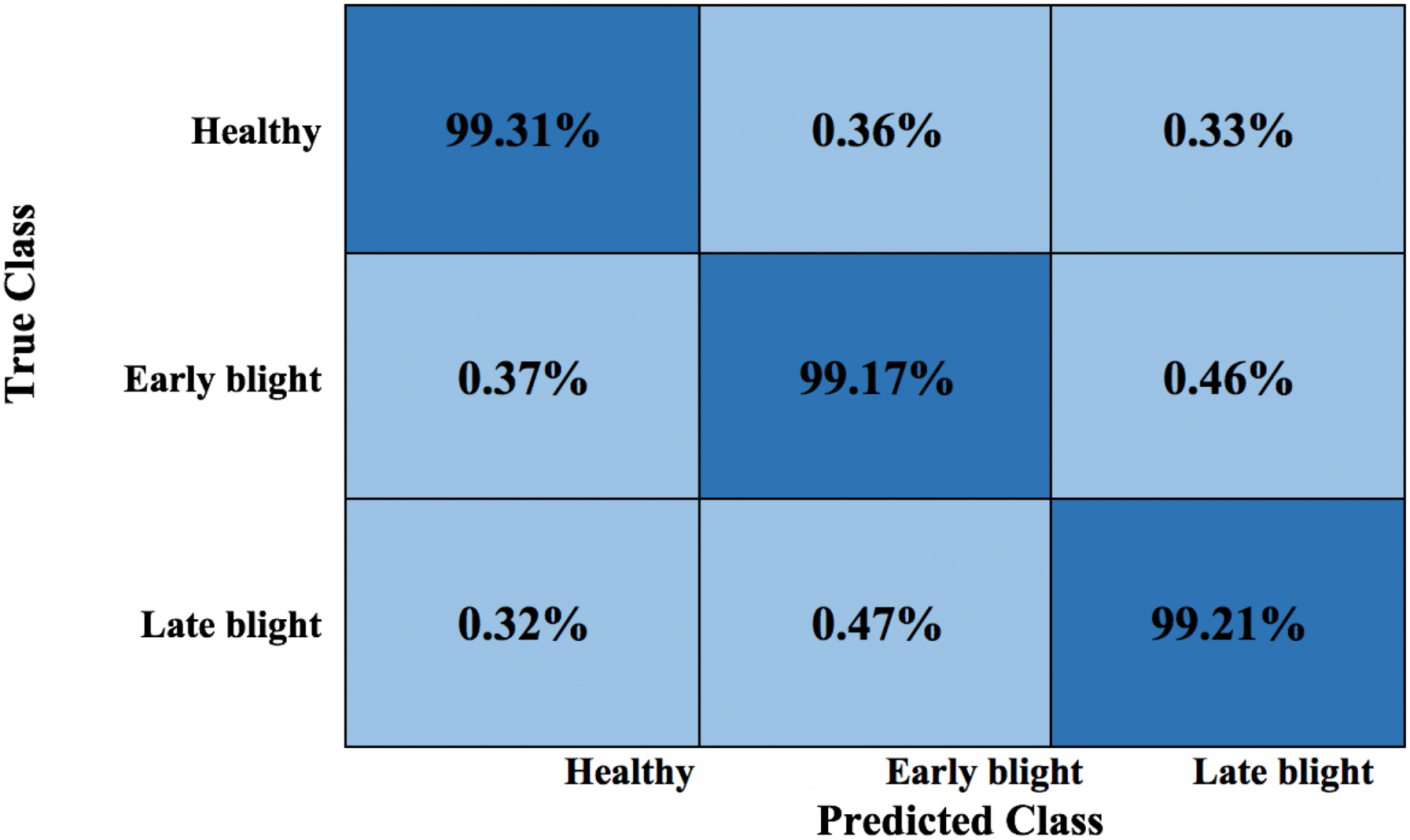
CM of the presented technique.

Moreover, the confusion matrix (CM) is reported in the context of plant leaf disease recognition; computing a CM is critically important, as it provides a granular valuation of the technique’s performance, enabling a detailed understanding of how well the model identifies different diseases. This is particularly significant in agriculture, where specific diseases can have varying impacts on crop health and productivity. By analyzing the CM, researchers and stakeholders can pinpoint areas where the model excels and where it falls short, facilitating targeted model improvements and resource allocation. The attained CM for the proposed method is given in **Figure 10**; from this, the scores demonstrate that effective results for all groups are attained. The largest error value is reported in the Late and early blight classes with a score of 0.47% due to textural resemblance among the infected regions of both groups; however, both groups are highly differentiable.

Finally, the scores for the entire dataset are reported, encompassing precision, recall, error rate, accuracy, and F1 measure to explain the overall performance of the presented technique for categorizing the various types of potato leaf diseases from healthy samples. **Table 1** depicts the performance analysis, which indicates that it performs well in terms of entire parameters. The suggested model has attained an average precision score of 99.36%, along with a recall of 99.23%. Further, this work accomplished an F1 score of 99.29% and an error rate of 0.71%. Moreover, the overall accuracy is 99.41%, which proves the efficacy of the proposed approach.

**Table 1:** Overall performance results of the proposed PotatoGuardNet approach.

Metric	Value (%)
Accuracy	99.41
Precision	99.36
Recall	99.23
F1-Score	99.29
Error Rate	0.71

### 4.6 Evaluation with base networks

This sub-section involves comparing and evaluating the proposed approach against other foundational techniques. Like RCNN (Bharati and Pramanik, 2020), Fast-RCNN (Girshick, 2015), Conventional Faster-RCNN (Sun et al., 2018), SSD (Liu et al., 2016), YOLO-V4 (Dewi et al., 2021), and YOLO-V4 tiny (Wang et al., 2021a) as given in (Wang et al., 2021b), which used the same PlantVillage potato leaf disease subset. In this work, we did not re-train these models under our experimental settings; instead, their originally reported accuracies were used for comparison. The main motivation for performing this experiment is to analyze the recognition ability of the suggested approach with the peer models. The performance comparison is performed with the employment of the accuracy metric, and the accomplished examination is offered in **Table 2**, which illustrates that the proposed method reaches the highest categorization outcomes as compared to other base approaches. From the comparison performed in **Table 2**, it is evident that the RCNN approach achieved the lowest accuracy score at 87.66%, followed by the Fast-RCNN approach with the second lowest score of 89.04%. Comparatively, the YOLO-V4 approach describes better performance with 95.18%. classification accuracy of 95.18%. In contrast, the proposed model demonstrates the highest results, achieving an accuracy score of 99.41%. Evidently, the base approaches have shown an average classification accuracy score of 93.58%, which is 99.41% for this case. Thus, the suggested model has provided a performance gain of 5.88%, which shows the robustness of the proposed method. The primary factor contributing to this effective classification outcome is that the RCNN and Fast-RCNN models rely on handcrafted features in their region proposal stage, which are incapable of capturing the complex patterns present in plant leaf images, potentially limiting the capability of method to accurately identify and categorization. Further, a conventional Faster-RCNN approach comprises shallower architecture, which limits its ability to fully capture the image information under complex background settings. Moreover, the SSD model involves a fixed set of default bounding boxes and does not adapt well to the diverse potato plant disease classes, potentially affecting its recognition ability. Further, the YOLO models struggle to handle the small, infected regions of plant leaves. In comparison, the suggested Inception-ResNetV2-based Faster-RCNN better tackles the issues of the existing models by offering effective feature-capturing capability, mitigating model overfitting, and balancing efficiency and accuracy. Based on the conducted analysis, it can be inferred that the proposed approach is more effective in identifying and grouping potato plant leaf diseases.

**Table 2:** Classification result comparison with base techniques.

Model	Accuracy (%)
RCNN	87.66
Fast-RCNN	89.04
Conventional Faster-RCNN	90.43
SSD	89.27
YOLO-V4	95.18
YOLO-V4 tiny	93.53
**PotatoGuardNet (Proposed)**	**99.41**

### 4.7 Analysis of DL approaches

This section demonstrates the conducted evaluation of the proposed method with several DL models like VGG-16 (Nawaz et al., 2022a), VGG-19 (Mateen et al., 2018), ResNet-34 (Koonce and Koonce, 2021), ResNet-101 (Nawaz et al., 2021), and DenseNet-121 (Nawaz et al., 2023) to compare the results against them. The comparison is explained in terms of accuracy measures and provided in **Table 3**. It can be visualized from the scores provided in **Table 3** that the proposed technique has outperformed the DL approaches with the highest classification score of 99.41%. The values in **Table 3** explain that the VGG-16 model exhibits the lowest performance among the models evaluated, with 96.81% due to its relatively shallow architecture, which hinders its ability to capture complex features in plant leaf images, potentially resulting in lower classification accuracy for fine-grained disease recognition. The second-lowest results are achieved by the ResNet-34 approach with a score of 97.03% because of a shallower structure, which prohibits it from intricate disease-related features in plant leaf images. Furthermore, the DenseNet-121 shows better results with a score of 98.50%. While reasonably, the proposed model shows the highest outcomes and provides a performance gain of 1.82% in comparison to all other methods. The superior recognition ability of the proposed approach can be attributed to its enhanced feature-capturing capability, which empowers it to tackle the complex background settings of the samples with a high recall rate. So, the proposed approach has provided a more robust framework in comparison to other DL models in recognizing the healthy and diseased samples of potato plant leaves.

**Table 3:** Classification result comparison with DL models.

Model	Accuracy (%)
VGG-16	96.81
VGG-19	97.80
ResNet-34	97.03
ResNet-101	97.83
DenseNet-121	98.50
**PotatoGuardNet (Proposed)**	**99.41**

### 4.8 Analysis with ML approaches

In addition, the results of the proposed approach are analyzed in comparison to various ML classifiers by comparing the accuracy value of the approach against them. For this purpose, well-known ML classifiers like SVM (Nawaz et al., 2022b), KNN (Wang et al., 2022), and RF (Das et al., 2023) have been nominated, and the attained comparison is provided in **Table 4**. The values in **Table 4** clearly show that this model has attained the highest accuracy in comparison to other ML approaches. The lowest score is accomplished by the RF, having an accuracy score of 91.08%, whereas the KNN achieved 93.32% accuracy, which is higher than the performance of SVM (95.82%), which is higher than the two methods. Comparatively, the proposed approach has attained the highest classification score of 99.41%. The motivation for this improved performance is that the RF classifier fails to handle the complex, non-linear relationships often present in image data, which results in reduced classification performance, particularly when dealing with intricate disease patterns on plant leaves. The KNN classifier cannot tackle high-dimensional image data, resulting in model over-fitting issues. Moreover, the SVM classifier is not proficient in tackling the multi-class classification issue. Comparatively, the proposed approach has better overcome the problems of these classifiers by proposing a highly effective feature selection and classification approach with a high recall rate, which empowers it to tackle the complex and distorted samples. The inclusion of Inception-ResNetV2 effectively tackles high-dimensional image data by using dimensionality reduction techniques, including factorized convolutions and IMs. These strategies help the model process intricate patterns within plant leaf images more efficiently. Additionally, Inception-ResNetV2’s incorporation of residual connections mitigates overfitting by promoting feature reuse, enhancing generalization, and enabling the model to perform well even on complex and diverse datasets.

**Table 4:** Classification result comparison with ML models.

Classifier	Accuracy (%)
SVM	95.82
KNN	93.32
RF	91.08
**PotatoGuardNet (Proposed)**	**99.41**

### 4.9 Analysis with state-of-the-art

To compare the proposed work with state-of-the-art techniques (Iqbal and Talukder, 2020; Mahum et al., 2023; Nazir et al., 2023; Kumari et al., 2025; Zhang et al., 2025), the analysis in **Table 5** is presented. In (Mahum et al., 2023), the authors proposed an improved DenseNet approach for classifying potato leaf diseases and accomplished 97.20% accuracy. Iqbal et al (Iqbal and Talukder, 2020). proposed an approach by employing the GLCM along with the RF classifier for categorizing the various types of potato plant leaves and attained a performance accuracy of 97%. Further in (Chen et al., 2023), a DL approach was suggested where the MobileNet approach was employed to calculate the key points from the given images and classify potato plant leaf diseases. This work (Chen et al., 2023) has accomplished an accuracy of 97.33%. Nazir et al (Nazir et al., 2023). suggested the DL approach by employing the EfficientNet in an end-to-end manner for classifying the various types of leaf diseases and reported 98.12% accuracy. Next, the work in (Zhang et al., 2025) trained 4 DL models named VGG16, MobileNetV1, ResNet50, and ViT, with VGG16 achieving the best accuracy. To improve efficiency, they proposed an enhanced model, VGG16, by restructuring the network, integrating the CBAM attention mechanism, and introducing Leaky ReLU. The work has attained an accuracy score of 95.82% over the PlantVillage dataset; however, at the expense of a huge computing burden. Next, Kumari et al (Kumari et al., 2025). proposed an ML approach for potato plant leaf classification, in which approaches like HT and DWT were used across different color spaces. The computed keypoints were classified using multiple ML predictors, with LR achieving the highest accuracy of 99% in the YCbCr color space. The work cannot locate the exact location of the diseased region. Moderately, the proposed method has scored the highest accuracy of 99.41%. In (Shaheed et al., 2023), a model named EfficientRMT-Net was suggested that combined Vision Transformer and ResNet-50 features, along with depth-wise convolution and stage-block structures, to competently extract and learn discriminative patterns from potato leaf images. This work has attained an accuracy of 99.12% over the PlantVillage dataset, however, at the expense of huge computing burden. Overall, the comparative methods have achieved an average score of 98.26%, which is 99.41% for the proposed model. So, the proposed model has attained a performance enhancement of 1.15% as compared to its competitor approaches. The key reason for the improved classification result of the proposed technique is its effective visual information capability, which assists it in better identifying and classifying the diseased regions. Additionally, its resilience against overfitting enhances generalization, making it effective across diverse disease classes and environmental conditions, which provides it a performance edge as compared to its peer models.

**Table 5:** Comparative analysis with the latest approaches.

Reference	Technique	Accuracy (%)
(Mahum et al., 2023)	Efficient DenseNet	97.20
(Iqbal and Talukder, 2020)	GLCM, along with the RF classifier	97
(Chen et al., 2023)	MobileNet-V2	97.33
(Nazir et al., 2023)	EfficientNet-V2	98.12
(Zhang et al., 2025)	Improved VGG16	95.82
(Kumari et al., 2025)	HT + DWT + LR	99
(Shaheed et al., 2023)	EfficientRMT-Net	99.12
**PotatoGuardNet (Proposed)**	**Improved FasterNet**	**99.41**

### 4.10 Cross-dataset evaluation

To evaluate the robustness and real-world applicability of the proposed framework, a cross-dataset experiment was conducted. The model was trained exclusively on the PlantVillage dataset, which contains leaf images captured under controlled lighting conditions with uniform backgrounds, and subsequently evaluated on the PlantDoc dataset (Singh et al., 2020), a field-based dataset comprising images acquired under diverse environmental conditions, including variable illumination, cluttered backgrounds, occlusions, and natural noise. Despite the considerable domain shift between the two datasets, the proposed Inception-ResNetV2–based Faster-RCNN model achieved an accuracy of 72.45% on the PlantDoc dataset without any domain adaptation or fine-tuning. Although a performance gap is observed when compared to results obtained on controlled data, this outcome highlights the model’s sensitivity to real-world variations such as background complexity, lighting changes, and disease appearance. Nevertheless, the achieved performance on unseen field data indicates that the proposed framework learns disease-relevant visual features rather than dataset-specific artifacts, thereby demonstrating its generalization capability and potential suitability for real-world agricultural deployment.

## 5 Conclusion

This research underscores the critical importance of automated potato plant disease classification in modern agriculture. To accomplish this, an improved DL approach has been designed for the timely and reliable detection of various potato leaf diseases. The conventional Faster-RCNN approach has been modified by employing the InceptionResNet-V2 framework as the feature extractor. A vast experimental evaluation comprising various analyses with base, ML, DL, and the latest approaches has been carried out to show the effectiveness of the proposed approach by employing a complex data sample from the standard database called the PlantVillage dataset. Besides, the localized and heatmaps generated samples have been investigated to show the recall and recognition capabilities of the proposed method. According to the visual and numeric analysis, it can be said that the implementation of advanced computer vision techniques, i.e., the Inception-ResNetV2-based Faster R-CNN model, offers a promising solution for accurate and timely identification. Such technology not only can enhance crop management by mitigating misdiagnoses and delays but can also contribute to increasing crop productivity and reducing production costs. A key limitation of this study is the limited availability of suitable field datasets. Future research will prioritize evaluation on newly collected real-world agricultural data and the integration of domain adaptation methods to further strengthen generalization across diverse environments.

## Data Availability

The original contributions presented in the study are included in the article/supplementary material. Further inquiries can be directed to the corresponding authors.
